# An assessment of chromosomal alterations detected by fluorescence in situ hybridisation in pancreatobiliary tract malignancy

**DOI:** 10.1186/s12876-020-01439-0

**Published:** 2020-11-04

**Authors:** Xiaohong Pu, Hongwei Zheng, Xin Yang, Qing Ye, Zhiwen Fan, Jun Yang, Xiangshan Fan, Xiaoping Zhou, Yudong Qiu, Qin Huang, Hongyan Wu, Jun Chen

**Affiliations:** 1 Department of Pathology, The Affiliated Drum Tower Hospital of Nanjing University Medical School, 321 Zhongshan Road, Nanjing, 210008 Jiangsu China; 2 Imaging department, Henan Provincial Hospital, Northwest corner of intersection of Dongting Lake Road and Huaxia Avenue in Zhengzhou Airport Economic Comprehensive Experimental Zone, Zhengzhou, 450000 Henan China; 3 Department of Medical Genetics, Nanjing Medical University, Nanjing, 210008 Jiangsu Province China; 4 Department of Digestive Medicine, The Affiliated Drum Tower Hospital of Nanjing University Medical School, 321 Zhongshan Road, Nanjing, 210008 Jiangsu China; 5 Department of Hepatobiliary Surgery, The Affiliated Drum Tower Hospital of Nanjing University Medical School, 321 Zhongshan Road, Nanjing, 210008 Jiangsu China

**Keywords:** Pancreatobiliary tract malignancy, FISH, Cytology, Paraffin-embedded tissue

## Abstract

**Background:**

Using fluorescence in situ hybridisation (FISH) to detect any gain of chromosomes 3, 7, or 17 and loss of the 9p21 locus has been proven to be sensitive in the diagnosis of pancreatobiliary tumors. However, both genetic and environmental factors contribute to the pathogenesis of pancreatobiliary tumors. Therefore, it is unknown whether this method is suitable for Chinese patients with pancreatobiliary tumors. This study aims to compare the sensitivity, specificity, predictive values and accuracy of cytology, ERCP/MRCP and FISH based on Chinese patients with pancreatobiliary tumors,and to analyze differences between brushing-based and formalin-fixed paraffin-embedded (FFPE)-based FISH.

**Methods:**

A total of 66 brush cytology specimens obtained during ERCP were detected by FISH and cytology test respectively to compare the sensitivity, specificity, predictive values and accuracy. Besides, FFPE-based FISH was performed on 46 corresponding paraffin sections of pancreatobiliary tumors obtained by surgical resection.

**Results:**

Our findings demonstrate that FISH greatly improves diagnostic sensitivity and negative predictive value compared to ERCP/MRCP and cytology without much reduction in specificity and positive predictive value. However, our results also indicate that FFPE-based FISH could not effectively identify the false-negative of brushing-based FISH.

**Conclusions:**

We believe that FISH can effectively distinguish true positive and false positive results of cytological or radiological suspicions of malignancy. However, FFPE-based FISH still does not precisely recognize the false-negative of brushing-based FISH. Both cytology-based and PPFE-based FISH had limitation in some specimens.

## Background

Pancreatobiliary tract carcinoma is a complex devastating disease with low survival rates and limited treatment options. Symptoms associated with pancreatobiliary tract malignancies include jaundice, itching and abdominal pain. Most of the patients seeking medical attention for these symptoms have been at advanced stage [1] and only 30% of patients have tumors resectable [2]. The 5-year survival rate for pancreatobiliary tract carcinoma is still less than 10% and many patients do not survive within 1 year after diagnosis [3].

Early accurate diagnosis with minimal invasiveness is critical for the treatment of pancreatobiliary tract carcinoma. However, the lack of reliable and accurate screening techniques greatly diminishes the chances of early detection. For now, diagnosis of pancreatobiliary tract malignancies is mainly based on clinical symptoms, radiology and cytology. Although there have been advances in non-invasive radiological techniques, it is still challenging to distinguish benign from malignant strictures through radiological imaging. Fibrotic biliary strictures induced by inflammation can mimic tumors. Furthermore, perihilar and distal cholangiocarcinomas tend to be periductal and seldomly mass-forming [4]. Although fine-needle aspiration (FNA) and endoscopic ultrasound-guided FNA have improved the diagnostic yield of biopsies, longitudinal tumor growth within a duct may be prone to sampling errors associated with FNA. Endoscopic or percutaneous FNA samples of the primary tumor also have limitations, such as collection of small, limited tissue samples without an intact mucosal layer, and possible seeding of malignant tumors, making FNA neither an ideal nor a recommended technique [5].

Brush cytology during endoscopic retrograde cholangiopancreatography (ERCP) has proven to be valuable in the assessment of perihilar, distal and pancreatic strictures under suspicion of malignancy. Brush cytology is the standard method used for the diagnosis of pancreatobiliary tract malignancies, which effectively obtains a complete biopsy sample without tumor seeding risk. A most important feature of brush cytology is its specificity, which can be as high as 100% [6]. However, its sensitivity is poor, which is reported from 6 to 64% [7]. Furthermore, its poor sensitivity is the main reason for the errors associated with sampling and cytological interpretation. Therefore, more accurate assays are needed to address these diagnostic challenges.

In this context, fluorescence in situ hybridisation (FISH) appears to be one of the most promising ancillary tests in clinical practice [8, 9]. More than 80% of cholangiocarcinomas contain chromosomal aberrations [10–12]. The use of FISH to detect DNA gains at chromosomes 3, 7 and 17 and loss at of the 9p21 locus has also been considered useful for the diagnosis of pancreatobiliary tumors [13, 14]. However, the pathogenesis of pancreatobiliary tumors is complicated, including genetic and environmental factors. Hepatitis virus infection is the main cause of cholangiocarcinoma in Chinese population, but primary sclerosing cholangitis is in western countries [15]. Therefore, it is not clear whether this technique is suitable for pancreatobiliary malignancy in Chinese population, although it has been used in the United States and other Western countries for decades. Consideration should be given to the re-identification of formalin-fixed paraffin-embedded (FFPE) samples, and whether such samples yield results of the same conditions as those of brushing samples.

Here, we first explored the sensitivity, specificity, predictive values and accuracy of FISH in brushing samples, and then further compared the consistency with the corresponding FFPE samples.

## Methods

### Study design

This study aims to compare all parameters including sensitivity, specificity, positive predictive value and negative predictive value between cytology, ERCP/MRCP and FISH based on pancreatobiliary tumors in Chinese populations, and to analyze the difference of brushing-based and FFPE-based FISH.

### Participants

**Fig. 1:**
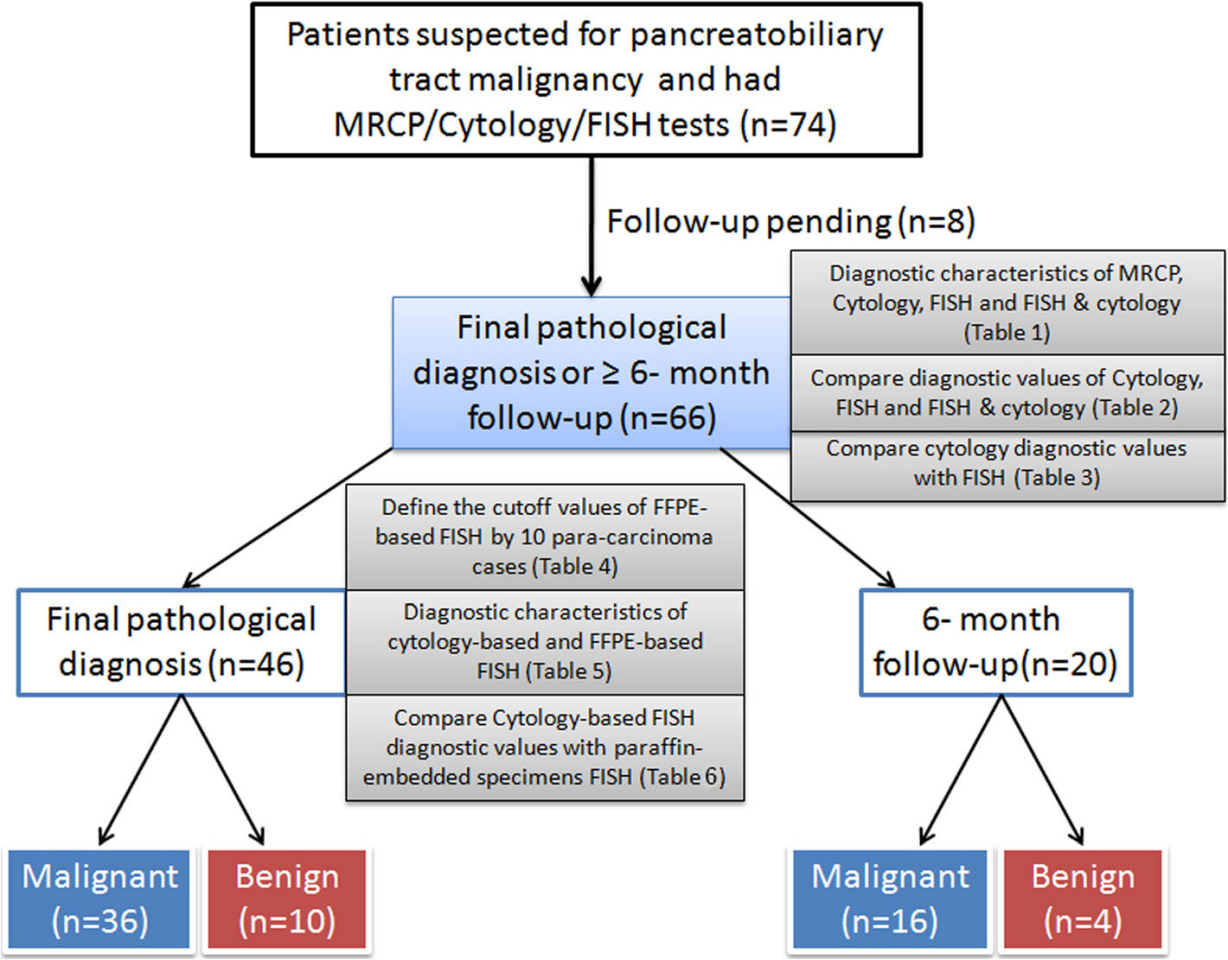
Study design. Seventy-four patients suspected for pancreatobiliary tract malignancy were screened by MRCP/ERCP, Cytology and FISH tests. Sixty-six patients had final pathological diagnosis or more than 6 months follow-up were included for comparing the diagnostic characteristics of ERCP, Cytology, FISH and FISH combined Cytology. Forty-six patients who had final pathological diagnosis were tested by PPFE-based and cytology brushing FISH to analyse the reason of false-negative in cytology-brushing specimens

From 2014 to 2017, 74 patients suspected of having pancreatobiliary tract malignancy, underwent ERCP, and had combined cytology and radiology, and FISH tests performed. Eight patients who had no pathological or follow-up records were excluded from the study. A diagnosis of malignancy was determined when final pathological results were obtained following surgery, or if radiological reports confirmed malignancy after 6 months. Diagnosis was considered benign after a ≥ 6-month follow-up or following confirmation by pathological results (Fig. 1).

### Test methods

#### Cytology

Specimens for both cytology and FISH were collected during ERCP with at least 10 to-and-fro motions at the site of the dominant stricture. The brushing specimens were placed in 15 mL of ThinPrep CytoLyt solution (Marlborough, MA, USA), and then transferred to the cytogenetics laboratory within 24 h. Cytologic diagnoses were classified using the Papanicolaou Society of Cytopathology guidelines for pancreatobiliary cytology [16], as follows: (1) Negative: a negative cytologic sample is synonymous with the absence of malignancy and cellular atypia and contains adequate benign cellular material. (2) Atypical: an atypical cytologic sample contains cells with morphologic features beyond normal changes but are insufficient to classify them as suspicious/malignant. (3) Suspicious: a suspicious cytologic sample contains cells with morphologic features that quantitatively and/or qualitatively fall short of a definitive diagnosis of malignancy. (4) Malignant/positive: a malignant cytologic sample contains cells that show malignant cytologic features. The categories of negative, atypical, suspicious, and malignant classification has been a standard practice in our laboratory since the guidelines were published in 2014. All of the cases were interpreted by a board-certified, experienced cytopathologist.

#### FISH in biliary brushing specimens

Samples were obtained using a separate standard cytology brush, following the collection of cytology specimens. After pre-treatment with phosphate buffered saline (PBS) and 0.075 mol/L potassium chloride at 37 °C for 10 min, cells were fixed with freshly made 3:1 methanol-acetic acid. Cytospin (Anbiping, Guangzhou, China) slides were prepared and stored at room temperature (RT). After re-fixation in 1% formaldehyde and PBS at room temperature, the slides were dehydrated in 70, 80 and 100% ethanol. We used a two probe mix, as opposed to the Vysis tetrachromic probe. One probe-mix contained three satellite-bound centromere-specific probes for chromosomes 3 (green), 7 (aqua) and 17 (red), and another contained a locus-specific probe for the locus 9p21 (P16, red) and chromosome 3 (green). There were two sets of each sample (chromosomes 3, 7 and 17; locus 9p21 and chromosome 3). After the slides were dried, a two probe-mix (Anbiping, Guangzhou, China) was placed on each target. Codenaturation (5 min at 73 °C) and hybridisation at 37 °C were carried out overnight in a Hybridizer (DAKO, California, USA), followed by a post-wash with 0.4× saline-sodium citrate (SSC) and 2× SSC. Diamidinophenylindole II was used as a counterstain. Slides were scored for hybridisation signals using the Olympus BX 51 Microscope (Olympus, Japan).

**Fig. 2:**
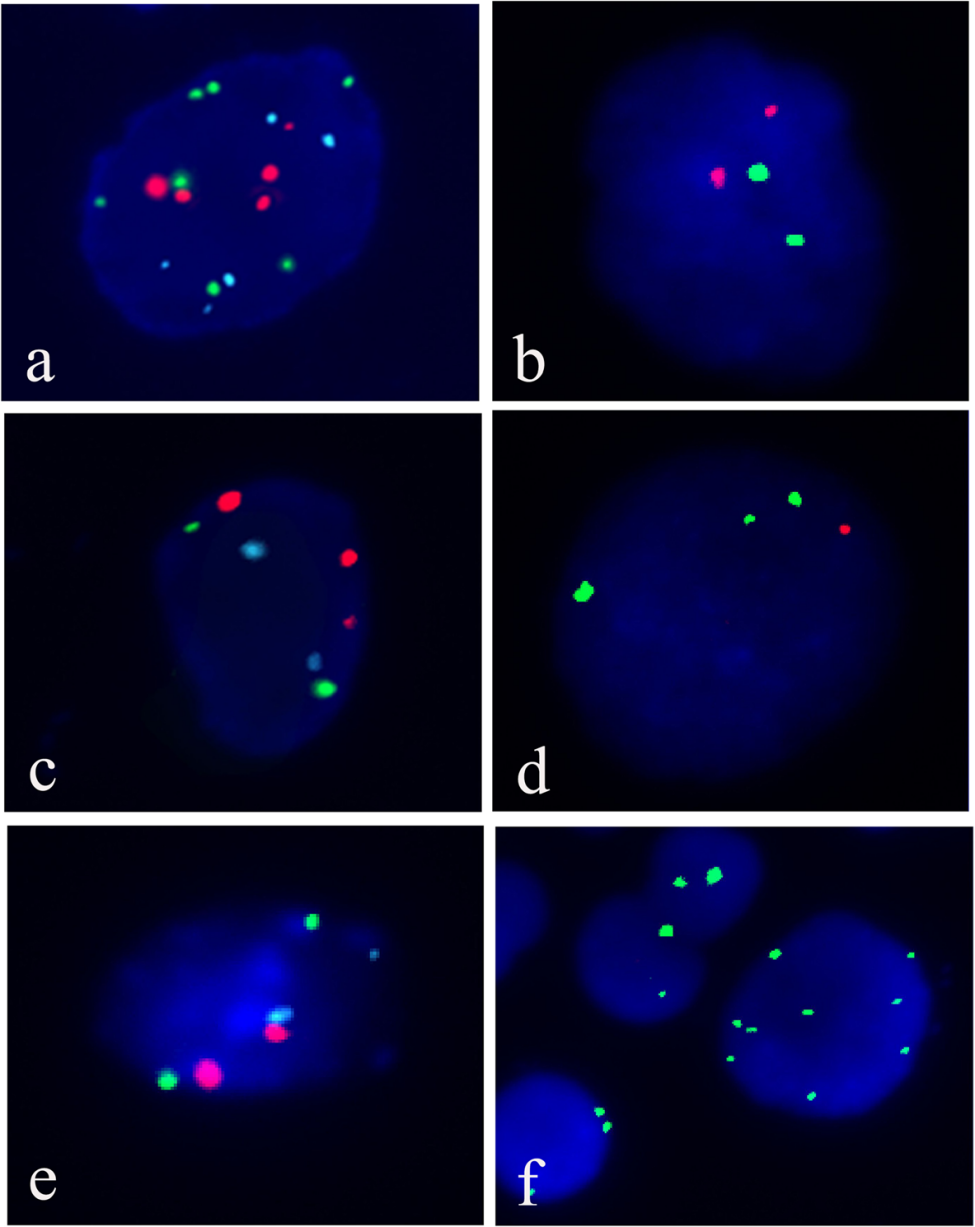
The positive criteria of FISH**.** Four or more cells with DNA gains at chromosomes 3, 7, and 17 (**a**) with or without loss of locus 9p21(**b**); more than four cells with polyploidy of only one chromosome (**c**) accompanied by loss of 9p21(**d**); and more than 12 cells with loss of 9p21(**f**) without gain of chromosomes 3, 7, and 17(**e**). Normal diploid cells were considered negative (**e** and **b**). Green signals for chromosome 3, aqua signals for 7, red signals for chromosome 17 in trichromic set and green signals for chromosome 3, red signals for 9p21 in double chromic set

Enumeration and evaluation of the FISH signals were performed on target cells that appeared morphologically abnormal. Positive criteria included: four or more cells with DNA gains at chromosomes 3, 7, and 17, or locus 9p21; more than four cells with polyploidy of only one chromosome, accompanied by loss of 9p21; and more than 12 cells with loss of 9p21. Normal diploid cells were considered negative (Fig. 2).

#### FISH in paraffin-embedded tissue samples

The FISH procedure was performed on 2-um thick formalin-fixed, paraffin-embedded tissue sections. Before hybridisation, slides were deparaffinised, dehydrated in 100% ethanol, and air-dried. Sections were digested in 5 mg/mL, pH 2.0 pepsin for 5–50 min. They were then fixed in 1% formaldehyde and phosphate-buffered saline at RT, and then dehydrated in 70, 80 and 100% ethanol. Probes (as described above) were subjected to codenaturation (8 min at 78 °C) and hybridisation at 37 °C overnight in a Hybridizer (Dako, California, USA). The cutoff values of FFPE-based FISH is based in Table 4: polysomy of chromosome 3 (≥ 5% of cells present more than five signals), polysomy of chromosome 7 (≥ 5% of cells present more than four signals), polysomy of chromosome 17 (≥ 4% of cells present more than three signals), loss of 9p21 (more than 8% of cells present no signal), loss of heterozygosity of 9p21 (more than 25% of cells present one signal), and gain of 9p21 (≥4% of cells present more than four signals).. The criteria for abnormality included: the counting of 100 cells and observation of more than two signals to reach the cut-off values.

### Statistical analysis

In statistical analysis, a cytology specimen was considered positive when carcinoma cells were identified, while a specimen interpreted as benign, atypical or suspected was categorized as negative. Surgery specimens were considered positive when definite carcinoma was present. Cytology, FISH samples and the final pathological results obtained following surgery were interpreted by three different pathologists respectively, and they were blinded tothe results of each other. Data in Table 4 were continuous variables, presentedas mean and standard deviation. Frequency data in Tables 1 and 5 were presented as numbers and percentages, and compared using the Chi-squared testor Fisher’s exact test. When the total number of samples exceeds 40, Chi-squared test was used, otherwise Fisher’s exact test was used. Sensitivity, specificity, positive predictive value, negative predictive value, and accuracy with exact 95% confidence intervals were obtained, based on the binomial distribution. Conventional two-tailed tests were conducted for all statistical analyses at a significance level of 0.05.

**Table 1:** Diagnostic characteristics of MRCP, Cytology, FISH and FISH & cytology

Characteristic	MRCP	Cytology	FISH	FISH & cytology
Sensitivity	26.9%(14/52)	40.4%(21/52)	76.9%(40/52)	78.8%(41/52)
Specificity	50.0%(7/14)	100%(14/14)	92.9%(13/14)	92.9%(13/14)
PPV	66.7%(14/21)	100%(21/21)	97.6%(40/41)	97.6%(41/42)
NPV	15.6%(7/45)	31.1%(14/45)	52.0%(13/25)	54.2%(13/24)
AUC(95%CI)	‡0.385 (0.212–0.557)	*0.702 (0.573–0.831)	*0.849 (0.742–0.956)	*§0.859 (0.754–0.963)

Sensitivity = True positive/(True positive+False negative)

Specificity = True negative/(True negative+False positive)

PPV = True positive/(True positive+False positive)

NPV = True negative/(True negative+False negative)

*FISH & cytology* FISH combined cytology, *FISH* fluorescence in situ hybridization, *MRCP* endoscopic Retrograde Cholangiopancreatography, *PPV* positive predictive value, *NPV* negative predictive value, *AUC* area under the curve, *CI* confidence interval

*P <0.01 represents difference compared with the cytology alone diagnostic strategy

‡ *P* = 0.021 represents difference compared MRCP with the cytology diagnostic strategy

§*P* = 1.000 represents comparison between FISH and FISH combined cytology

## Results

### Paticipants

Seventy-four patients (48 males and 26 females) suspected of having pancreatobiliary tract malignancy underwent magnetic resonance cholangiopancreatography (MRCP) and ERCP. Samples from these patients were collected for cytology and FISH during the period of the study. Eight patients without a definitive pathological diagnosis or 6-month follow-up data were excluded from the study. A total of 46 patients underwent surgery, thereby definitively diagnosed, 36 of whom demonstrated malignancies. Twenty patients had a 6-month follow-up, 16 of whom demonstrated malignancies. As depicted in Fig. 1, we evaluated the sensitivity and specificity of MRCP or ERCP, Cytology and FISH in diagnosis of 66 suspected pancreatobiliary malignancies in order to compare sensitivity, specificity, predictive values and accuracy of these three methods (Tables 1 and 2). And further compared the diagnosis results between cytology and FISH based on the “golden standard” of pathological diagnosis (Table 3). The cut-off value for FISH abnormalities in FFPE samples was determined through 10 adjacent normal tissues (Table 4). We further compared the results of FFPE samples and cytology-brushing samples in 46 pathological resection samples, trying to find the reason of false-negative in cytology-brushing-based FISH (Tables 5 and 6).

**Table 2:** Compare diagnostic values of Cytology, FISH and FISH & cytology

Pathology result	Cytology		FISH		FISH &cytology	
	Postitive	Negative	Postitive	Negative	Postitive	Negative
Positive (*n* = 52)	21	31	40	12	41	11
Negative (*n* = 14)	0	14	1	13	1	13
Total (*n* = 66)	21	45	41	25	42	24

Any of FISH or cytology positive was regarded as positive of “FISH & cytology”

Cytology Sensitivity = cytology positive/pathology positive = 21/52(40.4%)

Cytology Specificity = cytology negative/ pathology negative = 14/14(100%)

FISH Sensitivity = FISH positive/pathology positive = 40/52(76.9%)

FISH Specificity = FISH negative/ pathology negative = 13/14(92.9%)

FISH &cytology Sensitivity = FISH &cytology positive/pathology positive = 41/52(78.8%)

FISH &cytology Specificity = FISH &cytology negative/pathology negative = 13/14(92.9%)

*FISH & cytology* FISH combined cytology

**Table 3:** Compare cytology diagnostic values with FISH

Cytological Diagnosis	Final Diagnosis+(*n* = 52)		Final Diagnosis -(*n* = 14)	
	FISH+	FISH-	FISH +	FISH -
Positive(*n* = 21)	20	1	0	0
Suspicious(*n* = 5)	5	0	0	0
Atypical(*n* = 15)	9	5	1	0
negative(*n* = 25)	6	6	0	13
Total(*n* = 66)	40	12	1	13

FISH+ represents FISH positive

FISH- represents FISH negative

Final Diagnosis+ represents malignant by pathological diagnosis

Final Diagnosis- represents benign by pathological diagnosis

**Table 4:** Define the cutoff values of FFPE-based FISH by 10 para-carcinoma cases

Chromosome	Average signals ±3 Std. (n)	Cutoff signals (n)	Heteroploid cells + 3 Std (%)	Cutoff Cells (%)
Chromosome3(gain of signals)	1.84 + 3*1.02	5	3.56 + 3*0.54	5
Chromosome7(gain of signals)	1.12 + 3*0.93	4	2.10 + 3*0.72	5
Chromosome17(gain of signals)	1.96 + 3*0.50	3	1.43 + 3*0.74	4
9p21(loss of two signals)	1.84–3*0.57	0	6.00 + 3*0.55	8
9p21(loss of one signal)	1.84–3*0.57	1	16.0 + 3*2.98	25
9p21(gain of signals)	1.84 + 3*0.57	4	2.23 + 3*0.56	4

**Table 5:** Diagnostic characteristics of cytology-based and FFPE-based FISH

Characteristics	Cytology-based FISH	paraffin-embedded specimens FISH
Sensitivity	77.8%(28/36)	80.6%(29/36)
Specificity	90.0%(9/10)	90.0%(9/10)
PPV	96.6%(28/29)	96.7%(29/30)
NPV	52.9%(9/17)	56.3%(9/16)
AUC(95%CI)	0.839 (0.702–0.976)	0.853 (0.719–0.987)

*P* = 1.000 represents comparison between Cytology-based FISH and paraffin-embedded specimens FISH

**Table 6:** Compare Cytology-based FISH diagnostic values with paraffin-embedded specimens FISH

Paraffin- embedded specimens FISH	Final Diagnosis+(*n* = 36)		Final Diagnosis -(*n* = 10)	
	Cytology-based FISH+	Cytology-based FISH-	Cytology-based FISH +	Cytology-based FISH -
Positive(*n* = 29)	28	1	1	0
negative(*n* = 17)	0	7	0	9
Total(*n* = 46)	28	8	1	9

### Test results

In diagnosing malignancy, cytology alone had a sensitivity and specificity of 40.4 and 100%, respectively. The MRCP had a sensitivity of 26.9% and specificity of 50%, which were lower than cytology. The FISH had a sensitivity and specificity of 76.9 and 92.9%, respectively. Combined cytology and FISH (considered positive if positive on either test) showed a 78.8% sensitivity, a 92.9% specificity, positive predictive value 97.6%, and negative predictive value 54.2%. The overall diagnostic accuracy (or the trade-off between sensitivity and specificity) was determined by calculating the area under an ROC curve (AUC) for cytology alone, versus MRCP, versus FISH, versus cytology combined with FISH. Cytology is more accurate than MRCP/ERCP (*p =* 0.021), FISH and cytology &FISH are more accurate than cytology alone (*p<0*.01). No significant differences were observed between FISH and cytology combined with FISH (Table 1). The accurate diagnostic values of Cytology, FISH and FISH & cytology were presented on Table 2. Atypical and suspicious cytological samples (20/66) were observed in a considerable number of cases. Among those 20 samples, 15 were categorised as FISH positive, 14 of which were identified as malignant and only one was benign, according to histological evaluation. The remaining five samples categorised as FISH negative were diagnosed as pancreatobiliary tract tumors by histological diagnosis. Only one positive case by cytology showed negative by FISH (Table 3).

To define the cut-offs of chromosomal abnormalities in tumor specimens, ten para-carcinoma specimens were assessed to determine the distribution of FISH signals in normal tissue. Para-carcinoma specimens were areas of normal bile duct epithelium in histologic sections that also contained tumors. The mean signals of normal bile duct epithelium for chromosomes 3, 7, 17, and locus 9p21 were 1.84, 1.12, 1.96 and 1.84, respectively. The average number of heteroploid nuclei for the four probes were 3.56, 2.10, 1.43 and 6.00, respectively. Based on the mean ± 3 standard deviations, we established the cut-off values of polysomy of chromosome 3 (≥ 5% of cells present more than five signals), polysomy of chromosome 7 (≥ 5% of cells present more than four signals), polysomy of chromosome 17 (≥ 4% of cells present more than three signals), loss of 9p21 (more than 8% of cells present no signal), loss of heterozygosity of 9p21 (more than 25% of cells present one signal), and gain of 9p21 (≥4% of cells present more than four signals) were established as well (Table 4). The percentage of cut-off values of loss of 9p21 is relatively high, largely because of the truncation of nuclei in paraffin-embedded tissue and incomplete hybridisation [17, 18]. The criteria for abnormality included: the counting of 100 cells and observation of more than two signals to reach the cut-off values. No normal tissue specimens with values that exceeded these criteria were included in the evaluation of normal values.

The overall accuracy rate of FISH was 80.3% (53/66) to test the chromosome abnormalities in brushing cells. There were still 12 false-negative cases and one false-positive cases based on final pathological diagnosis. As positive criteria had been set by pathological diagnosis, we firstly evaluated the FISH results in histological samples. As mentioned above, 46 samples had definitive pathological diagnosis, 36 of which demonstrated malignant and 10 was benign. As depicted in Tables 5 and 6, the results of FFPE-based FISH were similar to those of cytology-based FISH. Of the eight cytology-based false negative cases, only one confirmed positive in the corresponding histologic FISH. The false positive sample in cytology-based FISH was still positive in FISH of the corresponding tissue section. These results suggested that FFPE-based FISH was not more sensitive than FISH for brushing specimens.

## Discussion

ERCP/MRCP and cytology are the primary tools for detecting pancreatobiliary tract malignancy. Nevertheless, ERCP/MRCP has poor sensitivity (range 56.5–65.1%) in reported studies [19], and even lower (26.9%) in our institution. Although cytology has near-perfect clinical specificity, it has been plagued by low diagnostic sensitivity. A ten-year review of literatures in cytology from ERCP reported that the sensitivities ranging from 6 to 64%, an overall sensitivity of 41.6% ± 3.2% (99% CI) and negative predictive of 58.0% ± 3.2% (99% CI) to diagnose malignancy [6, 20]. In our study, the sensitivity of cytology was 40.4% and the negative predictive was 31.1%. Our findings clearly show that FISH has higher diagnostic accuracy than MRCP/ERCP and cytology, which was consistent with other related studies. It is reported that the clinical utility of FISH in the diagnosis of indeterminate pancreatobiliary tract malignancy showed the sensitivity ranging from 34 to 58% and the specificity ranging from 89 to 98% [8, 13, 14, 21]. In our study, the sensitivity of FISH was 76.9% which slightly higher than reported literature. The probes for FISH are usually the Vysis tetrachromic probes in previous studies, whereas we used two probe mixtures and tested two sets for each sample. Probes for Chromosomes 3, 7 and 17 are in one set and those for locus 9p21 and chromosome 3 in the other. For each set, we counted at least 100 cells and observed the status of chromosome 3 for twice to improve the accuracy. Besides, our results indicated that FISH is particularly useful for detecting cytology negative and atypical samples. In our study, of the 15 atypical cytological samples, 14 were definitively diagnosed as tumors and 9 were determined to be FISH positive. Of the 25 cytology negative samples, 12 were confirmed tumors, and six of which were confirmed by FISH. Only one tumor was not diagnosed positive by FISH but positive by cytology. As the FISH assay is designed to identify aneusomic cells, it is conceivable that routine cytology can identify malignant cells from non-aneuploid pancreatobiliary tumors, which are false negative in FISH results. As to cases which are both negative may due to the the shortage of brushing, which not yield tumor cells in the brushing samples. Our study shows that the combined sensitivity of positive cytology and polysomy FISH is slightly higher than FISH alone.

Although the sensitivity of cytology-based FISH can reach 76.9%, some malignant samples remained undetected by FISH. FFPE and cytology brushing-based FISH document consistent patterns of chromosomal alteration. Moreover, FISH in FFPE specimens were not more sensitive than that in cytology-brushing specimens. Of the eight false negative samples in cytology-based FISH, only one was tested positive in corresponding FFPE-based FISH. And the one false-positive case in cytology FISH was still positive in corresponding paraffin-embedded specimens. One important consideration is the fact that cytology and FISH failure represent an established limitation of pancreatobiliary brush samples. Even if all specimens have a sufficient number of cells to reach diagnostic criteria, tumor locations that are difficult to sample with a brush, might not yield a sufficient number of either epithelial cells or tumor cells. Another important consideration is the fact that a portion of pancreatobiliary tumors might be non-aneuploid with respect to chromosomes 3, 7 and 17 and locus 9p21. This might explain why seven pancreatobiliary tract malignant samples were still tested negative in FISH of paraffin-embedded specimens. The combination of new FISH probe sets 1q21, 7p12, 8q24 and 9p21 have identified cancer cells in pancreatobiliary tissue samples with 93% sensitivity and 100% specificity [22], which reflects a higher sensitivity than that of the probe set for chromosomes 3, 7 and 17 and locus 9p21.Furthermore, some false negative results could be attributed to the limited number of chromosomal aberrations. In the present study, some samples showed only one chromosomal aberration, which was insufficient to reach the set diagnostic criteria. For example, DNA gains at chromosome 7 represent the most common chromosomal gain. The epidermal growth factor receptor gene is located on chromosome 7 (7p12), and may be involved in the development of cholangiocarcinoma [23, 24]. Patients with FISH results of trisomy 7 were 1.8 times more likely to have cancer than those with a negative FISH result [20]. Future prospective studies may further narrow the number of probes required to diagnose cancer.

The only one false positive sample in cytology-based FISH in our study was obtained from a 62-year-old female patient, who presented with jaundice and abdominal pain. The ERCP-based cytology and MRCP showed no discernible problems, and there was no apparent disease progression after a 6-month follow-up. In addition, a liver biopsy revealed no abnormalities. DeHaan et al. reported polysomy in both cholangiocarcinoma and high-grade dysplasia (HGD) in a study of paraffin-embedded specimens [25]. High-grade dysplasia, which is the morphologic precursor to frank cholangiocarcinoma, was observed to have a level of genetic abnormality by FISH [26, 27]. It could take months, or even years, for patients with HGD to progress to cholangiocarcinoma, and in some cases this progression might not occur. Thus, diagnostic tests for a patient with HGD might be limited to biopsy, thereby reducing the chances of achieving a more precise diagnosis. We suggest that if polysomy is observed in patients, without definitive clinical findings of malignancy, the diagnosis should be interpreted more cautiously.

Analysis of our findings also suggests that FISH testing could be used to screen some specific malignancies, such as cholangiocarcinoma with neuroendocrine differentiation. For example, one 57-year-old male patient was found to have perihilar space-occupying lesions on MRCP, but could not be definitively diagnosed. Following cytological tests, atypical cells were observed malignant in following FISH testing. His definitive pathological diagnosis is moderately differentiated cholangiocarcinoma with neuroendocrine differentiation.

## Conclusions

The evaluation of endoscopic brushings by FISH plays an important role in diagnosis of pancreatobiliary malignancy following routine cytology. The FISH presents a favourable option, with which the cytological suspicion of malignancy can be confirmed. By evaluating paraffin sections of pancreatobiliary tract malignancies with FISH, we further supported its validity as a diagnostic tool for brushing specimens. Further prospective studies might be necessary to define a more precise diagnostic algorithm. Consideration should also be given to aneuploidy of other chromosomal regions, to increase diagnostic yield and sensitivity.

## Data Availability

The datasets used and/or analysed during the current study are available from the corresponding author on reasonable request.

## Abbreviations

ERCP: Endoscopic retrograde cholangiopancreatography
FISH: Fluorescence in situ hybridisation
FNA: Fine-needle aspiration
HGD: High-grade dysplasia
MRCP: Magnetic resonance cholangiopancreatography
PBS: Phosphate buffered saline
FFPE: Formalin-fixed paraffin-embedded
PSC: Primary sclerosing cholangitis
SSC: Saline-sodium citrate

## References

[1] Siegel R, Ma J, Zou Z, Jemal A (2014). Cancer statistics, 2014. CA Cancer J Clin.

[2] Kim CA, Bowie JU (2003). SAM domains: uniform structure, diversity of function. Trends Biochem Sci.

[3] Everhart JE, Ruhl CE (2009). Burden of digestive diseases in the United States part III: liver, biliary tract, and pancreas. Gastroenterology.

[4] Siegel R, Naishadham D, Jemal A (2013). Cancer statistics, 2013. CA Cancer J Clin.

[5] Blechacz B, Komuta M, Roskams T, Gores GJ (2011). Clinical diagnosis and staging of cholangiocarcinoma. Nat Rev Gastroenterol Hepatol.

[6] Volmar KE, Vollmer RT, Routbort MJ, Creager AJ (2006). Pancreatic and bile duct brushing cytology in 1000 cases: review of findings and comparison of preparation methods. Cancer.

[7] Draganov PV, Chauhan S, Wagh MS, Gupte AR, Lin T, Hou W, Forsmark CE (2012). Diagnostic accuracy of conventional and cholangioscopy-guided sampling of indeterminate biliary lesions at the time of ERCP: a prospective, long-term follow-up study. Gastrointest Endosc.

[8] Vlajnic T, Somaini G, Savic S, Barascud A, Grilli B, Herzog M, Obermann EC, Holmes BJ, Ali SZ, Degen L (2014). Targeted multiprobe fluorescence in situ hybridization analysis for elucidation of inconclusive pancreatobiliary cytology. Cancer Cytopathol.

[9] Kipp BR, Barr Fritcher EG, Pettengill JE, Halling KC, Clayton AC (2013). Improving the accuracy of pancreatobiliary tract cytology with fluorescence in situ hybridization: a molecular test with proven clinical success. Cancer Cytopathol.

[10] Mahlamaki EH, Barlund M, Tanner M, Gorunova L, Hoglund M, Karhu R, Kallioniemi A (2002). Frequent amplification of 8q24, 11q, 17q, and 20q-specific genes in pancreatic cancer. Genes Chromosomes Cancer.

[11] Schleger C, Arens N, Zentgraf H, Bleyl U, Verbeke C (2000). Identification of frequent chromosomal aberrations in ductal adenocarcinoma of the pancreas by comparative genomic hybridization (CGH). J Pathol.

[12] Shiraishi K, Okita K, Kusano N, Harada T, Kondoh S, Okita S, Ryozawa S, Ohmura R, Noguchi T, Iida Y (2001). A comparison of DNA copy number changes detected by comparative genomic hybridization in malignancies of the liver, biliary tract and pancreas. Oncology.

[13] Kipp BR, Stadheim LM, Halling SA, Pochron NL, Harmsen S, Nagorney DM, Sebo TJ, Therneau TM, Gores GJ, de Groen PC (2004). A comparison of routine cytology and fluorescence in situ hybridization for the detection of malignant bile duct strictures. Am J Gastroenterol.

[14] Barr Fritcher EG, Kipp BR, Halling KC, Clayton AC (2014). FISHing for pancreatobiliary tract malignancy in endoscopic brushings enhances the sensitivity of routine cytology. Cytopathology.

[15] Tyson GL, El-Serag HB (2011). Risk factors for cholangiocarcinoma. Hepatology.

[16] Pitman MB, Centeno BA, Ali SZ, Genevay M, Stelow E, Mino-Kenudson M, Castillo CF, Schmidt CM, Brugge WR, Layfield LJ (2014). Standardized terminology and nomenclature for pancreatobiliary cytology: the Papanicolaou Society of Cytopathology Guidelines. Cytojournal.

[17] Qian J, Bostwick DG, Takahashi S, Borell TJ, Brown JA, Lieber MM, Jenkins RB (1996). Comparison of fluorescence in situ hybridization analysis of isolated nuclei and routine histological sections from paraffin-embedded prostatic adenocarcinoma specimens. Am J Pathol.

[18] Ventura RA, Martin-Subero JI, Jones M, McParland J, Gesk S, Mason DY, Siebert R (2006). FISH analysis for the detection of lymphoma-associated chromosomal abnormalities in routine paraffin-embedded tissue. J Mol Diagn.

[19] Yeo SJ, Cho CM, Jung MK, Seo AN, Bae HI (2019). Comparison of the diagnostic performances of same-session endoscopic ultrasound- and endoscopic retrograde cholangiopancreatography-guided tissue sampling for suspected biliary strictures at different primary tumor sites. Korean J Gastroenterol.

[20] Fritcher EG, Kipp BR, Halling KC, Oberg TN, Bryant SC, Tarrell RF, Gores GJ, Levy MJ, Clayton AC, Sebo TJ (2009). A multivariable model using advanced cytologic methods for the evaluation of indeterminate pancreatobiliary strictures. Gastroenterology.

[21] Bangarulingam SY, Bjornsson E, Enders F, Barr Fritcher EG, Gores G, Halling KC, Lindor KD (2010). Long-term outcomes of positive fluorescence in situ hybridization tests in primary sclerosing cholangitis. Hepatology.

[22] Barr Fritcher EG, Voss JS, Brankley SM, Campion MB, Jenkins SM, Keeney ME, Henry MR, Kerr SM, Chaiteerakij R, Pestova EV (2015). An optimized set of fluorescence in situ hybridization probes for detection of Pancreatobiliary tract Cancer in cytology brush samples. Gastroenterology.

[23] Gwak GY, Yoon JH, Shin CM, Ahn YJ, Chung JK, Kim YA, Kim TY, Lee HS (2005). Detection of response-predicting mutations in the kinase domain of the epidermal growth factor receptor gene in cholangiocarcinomas. J Cancer Res Clin Oncol.

[24] Yoon JH, Gwak GY, Lee HS, Bronk SF, Werneburg NW, Gores GJ (2004). Enhanced epidermal growth factor receptor activation in human cholangiocarcinoma cells. J Hepatol.

[25] DeHaan RD, Kipp BR, Smyrk TC, Abraham SC, Roberts LR, Halling KC (2007). An assessment of chromosomal alterations detected by fluorescence in situ hybridization and p16 expression in sporadic and primary sclerosing cholangitis-associated cholangiocarcinomas. Hum Pathol.

[26] Brankley SM, Wang KK, Harwood AR, Miller DV, Legator MS, Lutzke LS, Kipp BR, Morrison LE, Halling KC (2006). The development of a fluorescence in situ hybridization assay for the detection of dysplasia and adenocarcinoma in Barrett's esophagus. J Mol Diagn.

[27] Fritcher EG, Brankley SM, Kipp BR, Voss JS, Campion MB, Morrison LE, Legator MS, Lutzke LS, Wang KK, Sebo TJ (2008). A comparison of conventional cytology, DNA ploidy analysis, and fluorescence in situ hybridization for the detection of dysplasia and adenocarcinoma in patients with Barrett's esophagus. Hum Pathol.

